# Supplementary material for: Genetic comparisons of interleukin-17 reveal a framework for complex signaling evolution
Source: bioRxiv. 2026 Apr 14:2026.04.13.718218. Preprint. [Version 1] doi: 10.64898/2026.04.13.718218 (PMC13105070; doi:10.64898/2026.04.13.718218)
Supplement: Supplement 3 [file media-3.zip › Supplemental files for paper/ERC/IL17xIL17_comparisons/IL17RD_IL17RB.pdf]

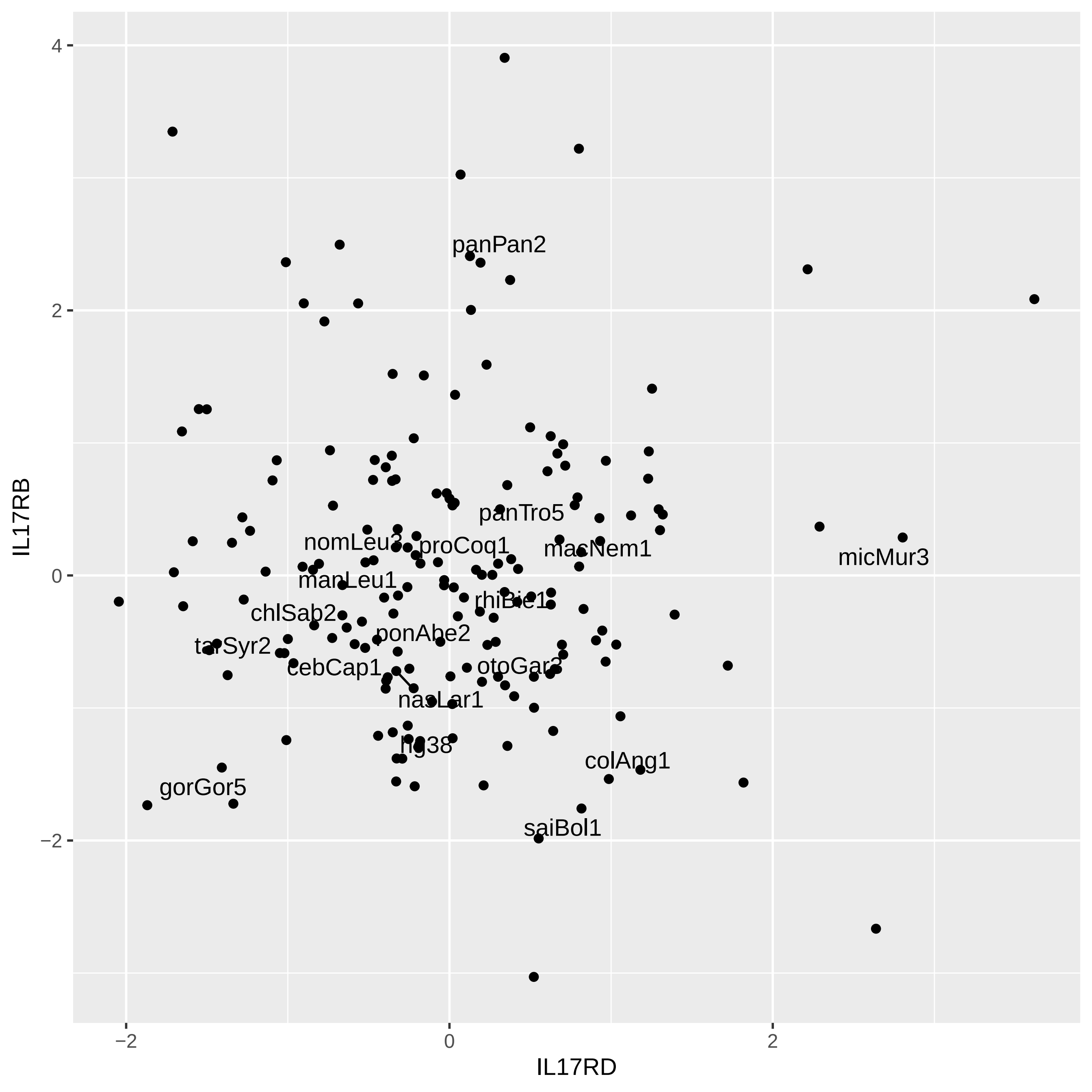

panPan2
panTro5
nomLeu3
proCoq1
macNem1
micMur3
manLeu1
rhiBie1
chlSab2
ponAbe2
tarSyr2
cebCap1
otoGar3
nasLar1
hg38
colAng1
gorGor5
saiBol1
IL17RB
IL17RD
4
2
0
−2
−2
0
2
